# H3^K27^-Altered Diffuse Glioma of the Spinal Cord in Adult Patients: A Qualitative Systematic Review and Peculiarity of Radiological Findings

**DOI:** 10.3390/jcm13102972

**Published:** 2024-05-18

**Authors:** Anna Maria Auricchio, Giovanni Pennisi, Grazia Menna, Alessandro Olivi, Marco Gessi, Gerrit H. Gielen, Simona Gaudino, Nicola Montano, Fabio Papacci

**Affiliations:** 1 Department of Neurosurgery, Fondazione Universitaria Policlinico Gemelli, 00168 Rome, Italy; anna.maria90a@gmail.com (A.M.A.); mennagrazia@gmail.com (G.M.); alessandro.olivi@policlinicogemelli.it (A.O.); nicolamontanomd@yahoo.it (N.M.); fabio.papacci@policlinicogemelli.it (F.P.); 2 Department of Neurosurgery, Università Cattolica del Sacro Cuore, 00136 Rome, Italy; 3 Department of Neurosurgery, F. Spaziani Hospital, 03100 Frosinone, Italy; 4 Department of Pathology, Fondazione Universitaria Policlinico Gemelli, 00168 Rome, Italy; mgessimd@yahoo.com; 5 Department of Neuropathology, Universitätsklinikum Bonn, 53127 Bonn, Germany; gerritgielen@web.de; 6 Department of Radiology, Fondazione Universitaria Policlinico Gemelli, 00168 Rome, Italy; simona.gaudino@policlinicogemelli.it

**Keywords:** spinal cord, H3^K27M^, diffuse glioma, radiological features

## Abstract

**Background:** Primary spinal cord diffuse gliomas (SpDG) are rare tumors that may harbor, like diffuse intrinsic pontine gliomas (DIPG), H3^K27M^ mutations. According to the WHO (2021), SpDGs are included in diffuse midline H3K27-altered gliomas, which occur more frequently in adults and show unusual clinical presentation, neuroradiological features, and clinical behavior, which differ from H3 G34-mutant diffuse hemispheric glioma. Currently, homogeneous adult-only case series of SpDG, with complete data and adequate follow-up, are still lacking. **Methods:** We conducted a qualitative systematic review, focusing exclusively on adult and young adult patients, encompassing all studies reporting cases of primitive, non-metastatic SpDG with H3^K27^ mutation. We analyzed the type of treatment administered, survival, follow-up duration, and outcomes. **Results:** We identified 30 eligible articles published between 1990 and 2023, which collectively reported on 62 adult and young adult patients with primitive SpDG. Postoperative outcomes were assessed based on the duration of follow-up, with outcomes categorized as either survival or mortality. Patients who underwent surgery were followed up for a mean duration of 17.37 months, while those who underwent biopsy had a mean follow-up period of 14.65 months. Among patients who were still alive, the mean follow-up duration was 18.77 months. The radiological presentation of SpDG varies widely, indicating its lack of uniformity. **Conclusion:** Therefore, we presented a descriptive scenario where SpDG was initially suspected to be a meningioma, but was later revealed to be a malignant SpDG with H3^K27M^ mutation.

## 1. Introduction

Spinal gliomas represent about 10% of all central nervous system (CNS) gliomas. [1] Ependymomas and pilocytic astrocytoma are the most common glial tumors in the spinal cord; in contrast, spinal cord diffuse gliomas (SpDG) are relatively uncommon [2]. Previously, SpDGs were categorized as IDH-wildtype tumors and typically harbored the *H3F3A-^K27M^* mutation, akin to other intracerebral diffuse midline gliomas (DMGs) [3,4]. These entities were noted to exhibit clinical behavior that differed from the aggressive nature typically observed in intracerebral DMGs *H3F3A-^K27M^* (e.g., pediatric diffuse-intrinsic pontine glioma) [3,5,6,7]. Consequently, with the latest update to the WHO classification in 2021 [4], SpDG H3^K27M^ mutants have been reclassified under diffuse midline H3K27-altered gliomas. This classification encompasses various types of H3^K27M^ mutations (vs. H3.3 G34-mutant of diffuse hemispheric glioma) and includes those lacking in IDH1/2. Furthermore, SpDGs can exhibit heterogeneous features in both clinical presentation and neuroradiological appearance [8,9,10,11,12]. This variability can present significant challenges in surgical planning and postoperative management. In our qualitative systematic review, focusing exclusively on the adult and young adult population, we aim to provide an overview of the descriptive course of SpDGs, including age distribution, spinal tract involvement, treatment modalities, duration of follow-up, and outcomes. Jung et al. [13] highlighted the diversity of MRI features observed in spinal cord diffuse midline gliomas with histone H3^K27M^ mutation, particularly in terms of longitudinal location and contrast enhancement. They noted that hemorrhage appeared to be the sole distinguishing feature between H3^K27M^-mutated tumors and wild-type ones. Our objective is to underscore this heterogeneity, exemplified by the atypical radiological presentation of an SpDG initially suspected to be a meningioma, but intraoperatively revealed as a malignant tumor.

## 2. Materials and Methods

### 2.1. Search Strategy and Selection of the Studies

An online literature search was launched on PubMed/Medline/Scopus/Google Scholar and Cochrane database using the following research string in any combination: “spinal cord” AND “diffuse glioma” AND “H3^K27M^” AND “adult”. The last research was conducted in February 2024.

We conducted a secondary search using the bibliographies of articles identified in our primary research. Articles were initially screened by title and abstract to assess their potential relevance. If the title and abstract did not indicate the degree of relevance, the full-text article was analyzed. All papers underwent independent review by two authors (A.M.A. and G.P.). Any discordance was solved by the consensus of the senior author (F.P.). Studies reporting the H3^K27M^ SpDG in adult and young adult patients were selected. The inclusion criteria were the availability of clinical and radiological reports for the single patients, human subjects, papers written in English or Italian language, and articles with the full-text available. We excluded guideline reviews, commentaries, and letters to the editor. According to the rarity of this pattern, case reports were also included.

### 2.2. Data Extraction

**Figure 1:**
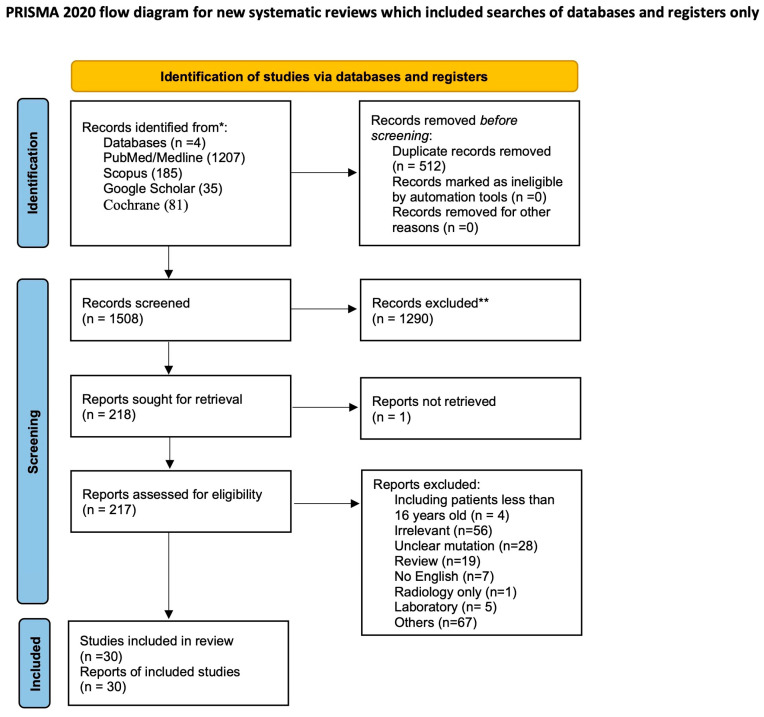
PRISMA flow chart. *: database; **: records excluded because they disagree with inclusion criteria.

According to the Preferred Reporting Items for Systematic Reviews and Meta-Analyses (PRISMA) statement (Figure 1), the following data were extracted: age, sex of patients with SpDG, grading and histopathology of H3^K27M^ mutation, spinal tract involved, type of treatment, and follow up. Complications were not reported in most studies and to avoid bias, they were not included in our data extraction record or statistical analysis. A total of 1508 records were found. A total of 30 papers satisfied our eligibility criteria, extracting 62 cases among only adult and young adult patients (≥16 years) and excluding pediatric cases. (Figure 1). To these data, we added our case of H3^K27M^ SpDG. The primary outcome of our systematic review was to determine the median follow-up duration and assess the status of patients (i.e., deceased or alive) at the time of follow-up, whenever this information was available.

### 2.3. Statistical Analysis

Quantitative variables were expressed as mean standard deviation and descriptive analysis was performed using Microsoft Excel v.16 (Microsoft, Redmond, WA, USA) and R software (version 4.0).

## 3. Results

### 3.1. Systematic Institutional and Literature Review

In our systematic review encompassing 62 patients (Table 1), there was no gender predominance (45% male and 45% female patients). The median age at diagnosis was 33 years (IQR 25–51), with the thoracic tract being the most common site of localization (29%), followed by the cervical tract (18%). Detailed histopathological data were available in 85% of cases. Surgery was performed in 44% of cases, while biopsy was conducted in 53% of cases. Chemotherapy and radiotherapy were administered in 43% and 50% of patients, respectively. Patients who underwent surgery had a mean follow-up of 17.37 months (SD ± 11.98), while those who underwent biopsy had a mean follow-up of 14.65 months (SD ± 11.89). For patients who were still alive, the mean follow-up duration was 18.77 months (SD ± 10.02). However, due to incomplete data, further analysis could not be conducted for several reasons:

− Histopathological records did not consistently meet c-IMPACt criteria for the diagnosis of DMG H3^K27M^ [6,14,15,16];
− Lesions were included according to both the WHO 2016 and WHO 2021 guidelines, depending on the publication date;
− Cases of diffuse and anaplastic astrocytoma were diagnosed according to the WHO 2016 criteria;
− Follow-up data were missing in 22 cases (35%), precluding calculation of the overall survival rate.

Among cases described as grade 2 or 3 SpDGs, the mean follow-up duration was 24.59 months (SD ± 11.55), compared to 15.76 months (SD ± 11.8) for cases diagnosed as spinal diffuse midline glioma, and 11.55 months (SD ± 10.11) for glioblastoma. The overall mean follow-up duration was 15.51 (SD ± 11.73) months. Notably, the longest follow-up durations were observed in patients with cervical and thoracic lesions, with mean durations of 17.95 months (SD ± 14.46) and 16.23 months (SD ± 8.13), respectively.

### 3.2. Unexpected Radiological Features in a Case of SpDG H3^K27M^

A 26-year-old man presented to the Emergency Department of Gemelli Hospital with a four-month history of recurring left shoulder pain, unresponsive to common non-steroidal anti-inflammatory drugs (NSAIDs). Over three weeks, his symptoms worsened, manifesting as persistent left hypoesthesia and dysesthesia, beginning from the left D1 root, with involvement of the ipsilateral shoulder, chest, and abdomen. Over the next 40 days, his symptoms worsened, developing mild left leg weakness, alterations in both deep and superficial sensory function, instability in gait, signs suggesting the pyramidal tract’s involvement, and neurological bladder dysfunction. Additionally, there was progressive weakening in the distal limbs, with a positive pronator drift test on the left side.

**Figure 2:**
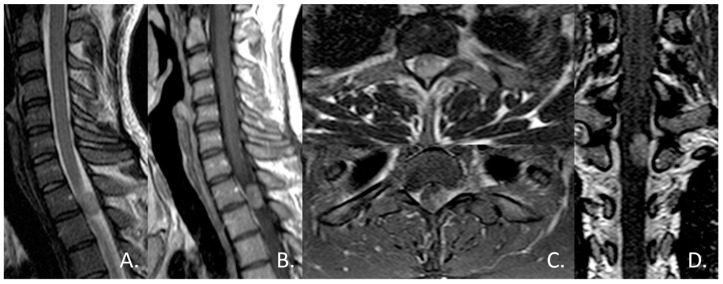
Sagittal T2-weighted (**A**) and contrast-enhanced T1-weighted (**B**) MR images showed a well-circumscribed, dorsally located, intradural extramedullary mass at the level of T1-T2, that caused anterior displacement of the spinal cord. Axial T2-weighted (**C**) and contrast-enhanced T1-weighted MR images confirmed the apparent location of the mass within the dural sheath, but outside the spinal cord, and coronal contrast-enhanced T1-weighted (**D**) better demonstrated the broad dural contact. The mass appeared as near iso-intensity to the spinal cord on T2, with homogeneous contrast enhancement.

An MRI scan revealed a well-defined, homogeneously enhancing extra-dural mass, located posteriorly within the vertebral canal at the T1–T2 level, causing significant cord compression (Figure 2). Although T2*-weighted images were not available to detect intra-tumoral bleeding, both T1-weighted and T2-weighted images exhibited a highly homogeneous signal. Furthermore, unlike other reported cases [13,14,17,18,19,20], there was no evidence of leptomeningeal enhancement on the initial MRI scan.

**Figure 3:**
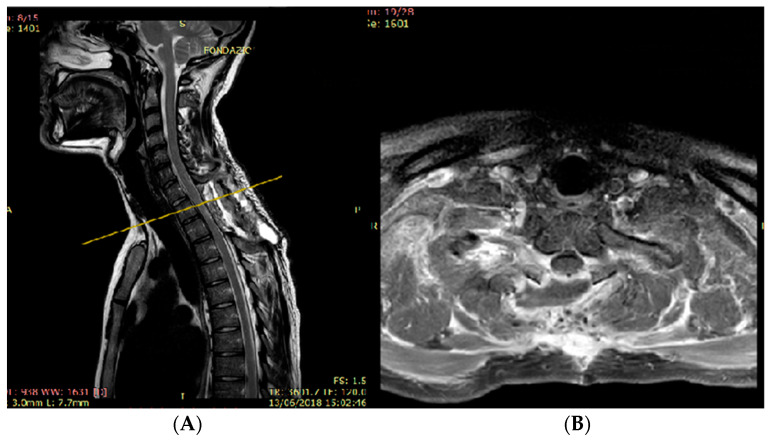
Sagittal T2-weighted MR image (**A**) showed the total resection of the mass and posterior paravertebral collection at the level of D1–D2, at the site of the laminectomy. Axial contrast-enhanced T1-weighted MR image (**B**) demonstrated the complete resection of the mass at the level of yellow line.

Initially, the tumor was diagnosed as a meningioma, based on MRI findings, which strongly suggested an extramedullary spinal tumor rather than an intrinsic medullary one. The imaging features were consistent with those expected for a meningioma, including the homogeneous signal, contrast enhancement, and a posterolateral location. The MRI’s differential diagnosis included nerve sheath tumors, ependymoma, or intradural metastases, but a glioma hypothesis was not considered. During surgery, the tumor appeared as an intramedullary exophytic lesion. A total resection was achieved under neurophysiological monitoring, which did not detect any variations in intraoperative motor potentials. Following surgery, the patient experienced relief from dorsal pain, and there was an improvement in his gait and leg weakness. Subsequent postoperative MRI revealed complete tumor removal (Figure 3).

**Figure 4:**
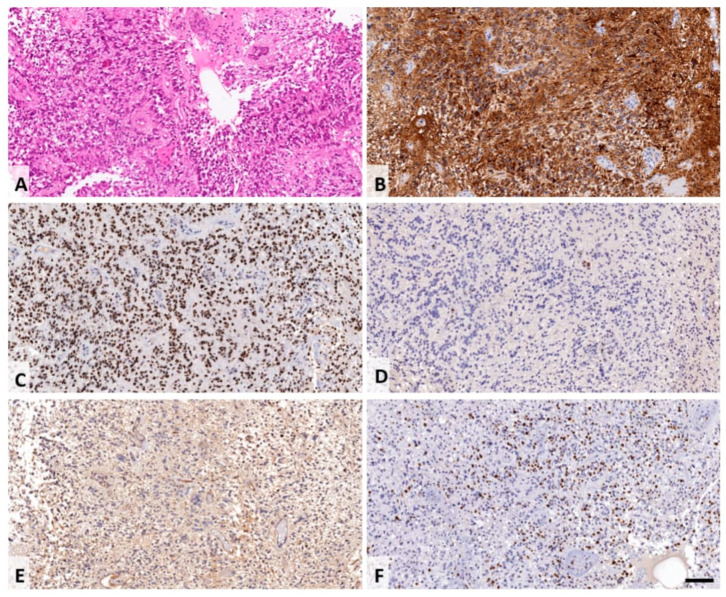
Histological findings of the resected tumor tissue, H and E staining shows fragments of a glial tumor, with areas of necrosis and vascular proliferations. Focally, the tumor displayed a perivascular arrangement of tumor cells resembling ependymoma-like pseudorosettes (**A**). Tumor cells were positive for glial fibrillary acidic protein (GFAP; (**B**)). A strong nuclear expression of H3^K27M^ mutant protein was found (**C**) and H3K27-3me expression was lost in tumor cells (**D**). Only few tumor cells expressed glial transcription factor Olig-2 (**E**). The proliferation index (Mib-1, (**F**)) labelled more than 10% of the tumor cells. Scale bar corresponds to 100 µm.

Histopathological analysis revealed multiple fragments of a glial tumor, with areas of necrosis and vascular proliferation. Notably, the tumor exhibited a perivascular arrangement of tumor cells, reminiscent of ependymoma-like pseudorosettes (Figure 4A, H and E stain). Tumor cells were positive for glial fibrillary acidic protein (GFAP; Figure 4B) and microtubule-associated protein 2 (MAP2); a “dot-like” pattern of epithelial membrane antigen (EMA) was focally observed, and a strong nuclear expression of H3^K27M^ mutant protein was found (Figure 4C), while H3K27-3me expression was lost in tumor cells (Figure 4D). In addition, only a few tumor cells expressed glial transcription factor Olig-2 (Figure 4E); p53 protein was expressed in more than 20% of the tumor cell nuclei, while the proliferation index (Mib-1, Figure 4F) labeled more than 10% of the tumor cells. The tumor did not show a clear loss of ATRX expression. Immunohistochemical analysis with antibodies against axonal neurofilament protein revealed an axonal structure within the tumor tissue, resembling a diffusely infiltrating glioma. The final diagnosis was spinal diffuse midline glioma (WHO 2016, grade IV), H3^K27M^ mutant.

Brain MRI results were unremarkable after surgery, and the patient was discharged without any acute complications.

However, one month later, the patient presented with a fluid puncture under the wound. Surgical revision revealed wound infection, which was successfully treated by a four-week course of antibiotic therapy, leading to complete resolution. After discharge, the patient was lost at follow-up.

**Figure 5:**
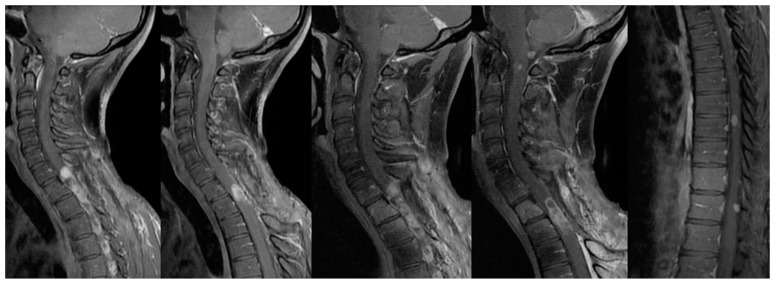
MRI at 11 months post-operation in sagittal contrast-enhanced T1-weighted images showing the progression of disease at different cervical tracts, and smaller additional intradural enhancing nodules along the surface of the tonsil of cerebellum, in the medulla oblongata, and in the lower thoracic spinal cord.

At 11 months post-operation, without any adjuvant chemo and radiotherapy, the patient came back to our department; a spinal MRI revealed tumor recurrence, with diffuse contrast enhancement along the spinal cord and surgical edges of the previous scar (Figure 5).

**Figure 6:**
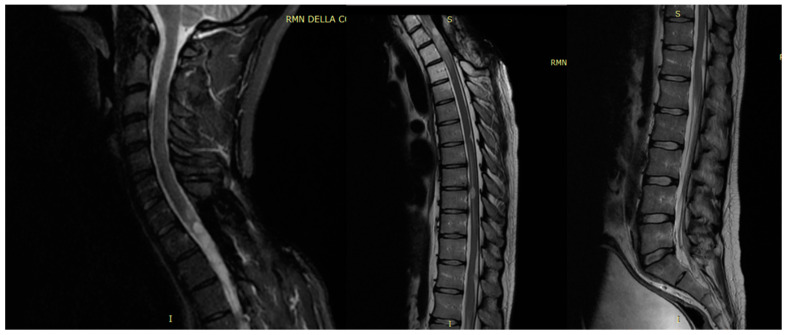
Follow-up spine MRI with disease progression in T2 sagittal view of the cervical, thoracic, and lumbar tracts after adjuvant therapy.

Consequently, the patient underwent two cycles of chemotherapy with temozolomide. Following completion of this protocol (3 months later), the patient reported paresthesia and hypoesthesia in both legs, ultimately becoming wheelchair-bound. Subsequent spine MRI revealed tumor progression. In response, the patient underwent a one-month cycle of Cyberknife treatment, delivering 45 Gy in 25 fractions, focusing on C6-D4 metameres. An extra dose of 50 Gy was administered to areas showing more enhancement, utilizing a 6 MeV linear accelerator with a modulated intensity kinetic conformal technique. This intervention resulted in clinical and radiological stability of the lesion for 5 months. However, a follow-up spine MRI showed disease progression with evidence of multiple enhancing nodules (Figure 6).

At the last follow-up available (20 months post-operation), the patient remained alive, awaiting a new cycle of palliative chemotherapy with temozolomide. All procedures adhered to the ethical standards outlined by the institutional research committee (Policlinico A. Gemelli) and the 1964 Helsinki Declaration, with written informed consent obtained from the patient for the publication of clinical details and images.

## 4. Discussion

SpDGs are rare tumors in adults, characterized by the presence of H3^K27M^ mutations, similar to other diffuse midline gliomas [3]. The presence of H3^K27M^ mutation has been reported not only in diffuse gliomas, but also in non-diffuse gliomas and glioneuronal tumors occurring in adult intracerebral midline tumors [5,11,12,21], but the biological significance of this mutational event is still debated. Conversely, in diffuse infiltrating tumors, the presence of the H3^K27M^ mutation has been predominantly associated with malignancy. However, according to the WHO 2016 classification, SpDGs with the H3^K27M^ mutation do not consistently exhibit an association with aggressive clinical behavior, as observed in patients with diffuse intrinsic pontine glioma (DIPG) harboring the H3^K27M^ mutation [3]. To better clarify this difference, the WHO 2021 classification categorizes these entities as diffuse midline H3K27-altered gliomas [4]. Due to the rarity of this pathology and the lack of large case series with homogeneous follow-up data, our focus was exclusively on adult and young adult patients, aiming to gather as much data as possible to initiate a descriptive analysis within this specific category. We hypothesized that the pediatric counterpart may exhibit more aggressive behavior, so it was excluded by the review [22]. The radiological findings in SpDGs can also pose challenges [13,22,23]. 

Therefore, presenting a case of a 26-year-old patient with thoracic SpDG H3^K27M^ and an unusually long survival of 20 months might be noteworthy, due to its atypical radiological features. In particular, to the best of our knowledge, this is the first case of SpDG H3^K27M^ presenting as an extra-dural lesion on MR imaging. This unique MRI appearance is more similar to extra-axial lesions, such as meningioma, and therefore less aggressive, which could have delayed surgery or have modified the therapeutic approach.

### 4.1. Systematic Review

A recent systematic review, performed by Watanabe in 2023 [2], included 39 studies with 279 patients. Despite there being no difference in age, the retrospective nature of many studies made data retrieval challenging, especially with regard to individual patient ages. We specifically focused on extracting data from adult and young adult cases (≥16 years old) to ensure clarity on patient age and follow-up duration [24]. We also included data from Lebrun et al. [25], focusing on adult patients identified as being >18 years old, due to the availability of follow-up information. Our analysis found that the median age (32 months) was consistent with the author’s findings, but in our review, no gender prevalence was detected. However, the mean overall follow-up duration in our study was 15.51 months (SD ± 11.73), compared to the reported mean overall survival duration of 24 months in the review by Watanabe [2]. This discrepancy may be influenced by inaccuracies in the mean overall survival data from the retrospective series, as evidenced by the lack of detailed descriptions and references for some cases (i.e., in the table there are five cases reported by Biczok in 2021, with no detailed description of data and with no reference available; Shows et al.’s report was not found, some case reports were missing and some data were reported as incorrect information) [2]. Therefore, we aimed to provide a more qualitative and accurate description of the available literature, as summarized in Table 2.

Due to the limited and sometimes unclear data, complete clinical or biological parameters for risk stratification and therapy optimization are still lacking. Therefore, enrollment in multicenter controlled trials is essential when encountering this rare entity for future research.

Various treatment combinations, including radiation therapy (RT) and chemotherapy (CT), were administered following surgical resection or biopsy. However, we found no significant difference in patient outcomes between surgical resection and biopsy in our review (*p* > 0.05). Specific treatment protocols varied among studies, with some authors describing radiation protocols:

- Schreck described a radiation protocol with a dose range starting from 36 Gray up to 65 Gray, with a most used dose interval of 46–55 Gy and 56–60 Gy [31];
- Other authors [27,31] described a therapy with radiation and concomitant adjuvant temozolomide, such as Morais et al. [1], who described spinal radiotherapy with 45 Gy in 28 fractions and chemotherapy with temozolomide (completed six cycles);
- Yi et al. [49] focused their attention on surgical strategy and RT and CT protocols, with no finding of statistical evidence in OS.

Additionally, histone deacetylase inhibitors such as valproic acid and palliative treatments like panobinostat/bevacizumab/irinotecan were reported [19,38,42]. Missing specific treatment details in a significant percentage of cases hindered the assessment of treatment impact on follow-up duration.

Furthermore, in the light of current evidence [2,49], gross total resection does not influence survival. Unfortunately, some missing specific details concerning all types of treatment, such as in 39% of lacking cases for chemotherapy and in 37% of cases for radiotherapy, do not allow for counting the impact of treatment on FU.

### 4.2. Radiological Finding

Among the few publications concerning SpDG H3^K27M^, those reporting MRI features are even fewer [13,23,50].

Jung et al. [13] reported the heterogeneity of MRI findings of spinal cord diffuse midline glioma with histone H3^K27M^ mutation, in terms of longitudinal location and contrast enhancement, and hemorrhage seemed to be the only feature to differentiate H3^K27M^-mutated tumors from wild-type ones. In the pediatric population, Hohm et al. [22] described SpDG H3.3 K27M mutant with cystoid necrosis and disomogeneous contrast enhancement with marked swelling of the conus medullaris and strong peripheral contrast enhancement. Lasocki et al. [23] defined the image features related to H3 K27-altered glioma as typical midline locations, with variable enhancement characteristics often less expected for a high-grade tumor. Leptomeningeal dissemination is frequently observed in the later stages of the disease course in H3^K27M^ SpDGs [23]. 

In our case, the tumor appeared as an intradural extramedullary mass on sagittal images, initially resembling primary diagnoses such as meningioma or nerve sheath tumors. On axial images, the relationship of the mass to the cord still suggested an intradural extramedullary location (i.e., within the theca, but outside the cord). Considerations should be made regarding the challenge of determining the compartment in which a larger lesion resides, especially when distinguishing between an extramedullary mass compressing the cord and an exophytic intramedullary mass. Additionally, the homogeneous signal intensity on T2 and the equally homogeneous contrast enhancement were reminiscent of a benign meningioma.

While H3^K27M^ SpDG has been reported to share MRI features with lower-grade gliomas, our case is the first to neuroradiologically resemble a low-grade extramedullary tumor. Therefore, this case highlights the importance of considering the possibility of H3^K27M^ SpDG presenting as an apparent extramedullary mass or an exophytic intramedullary lesion, especially in cases with rapidly worsening symptoms.

However, clinicians should maintain a high index of suspicion for H3^K27M^ SpDG, particularly when encountering cases with atypical clinical presentations or rapid neurological deterioration. This highlights the significance of comprehensive diagnostic evaluation, including molecular testing for H3^K27M^ mutation, to accurately diagnose and manage these rare tumors.

## 5. Conclusions

In conclusion, the biological significance of H3^K27M^ mutation in primary SpDGs in adult patients is not fully understood, but they probably differ from their highly aggressive intracerebral and pediatric counterparts. However, the rarity of such tumors hinders the definition of specific clinical studies and the development of tailored protocols for patients affected by H3^K27M^ SpDG.

Despite the limited data available on MRI features in H3^K27M^ SpDG in adults, even our single case, which differs from that reported so far in the literature, can help to expand our knowledge of the spectrum of MRI features in this tumor.

Varied approaches, including surgery, radiotherapy, and chemotherapy, with no significant difference in outcomes between surgical resection and biopsy need to be optimized to improve patient outcomes.

## Figures and Tables

**Table 1:** Characteristics of patients with H3^K27M^ spinal diffuse glioma in the literature.

Characteristics		N
**Total Patients**		62
**Male (%)**		28 (45%)
**Female (%)**		28 (45%)
**NA (%)**		6 (10%)
**Median Age (IQR) years**		33 (25–51)
**Grading**	2 (%)	3 (5%)
	3 (%)	5 (8%)
	4 (%)	54 (87%)
**Spinal tract**	Cervical (%)	11 (18%)
	Thoracic (%)	18 (29%)
	Lumbar (%)	2 (3%)
	Cervical-thoracic (%)	5 (8%)
	Thoracolumbar	5 (8%)
	Holocord	1 (2%)
	NA	20 (32%)
**Pathology**	Glioblastoma (%)	27 (43%)
	Diffuse midline glioma * (%)	22 (35%)
	Diffuse astrocytoma (%)	3 (5%)
	Anaplastic Astrocytoma (%)	10 (16%)
	Surgery (%)	27 (44%)
	Biopsy (%)	33 (53%)
	NA (%)	2 (3%)
**Treatment**	Ctx (%)	
	Yes	27 (43%)
	No	11 (18%)
	NA	24 (39%)
	Rtx (%)	
	Yes	31 (50%)
	No	8 (13%)
	NA	24 (37%)
**Mean Fu (SD±) months**	Overall	25.75 (SD ± 16.78)

DMG *: Diffuse midline glioma according to WHO 2016, or diffuse midline H3K27-altered glioma according to the WHO, 2021.

**Table 2:** Cases with spinal diffuse glioma with H3K27 mutation in adult and young adult patients.

Authors	Year	Gender	Age	Grading (WHO) *	Spinal Tract	Treatment	FU (Months)	Status	CTx	RT (y = Y; n = N)	Pathology
Morais et al. [1]	2012	M	19	4	Th	S	18	A	Y	Y	GBM
Gessi et al. [26]	2015	F	17	4	NA	S	NA	NA	NA	NA	GBM
		M	20	3	Th	S	NA	NA	NA	NA	An Astro
		M	22	4	Th	S	NA	NA	NA	NA	GBM
		M	34	4	Th	S	NA	NA	NA	NA	GBM
		M	55	4	L	S	NA	NA	NA	NA	GBM
		M	51	4	Th	S	NA	NA	NA	NA	GBM
		M	62	3	C	S	NA	NA	NA	NA	An Astro
		M	47	2	C	S	NA	NA	NA	NA	Diff Astro
Meyronet et al. [27]	2017	F	19	4	NA	S	1.5	D	N	N	GBM
		M	20	4	NA	B	7	D	Y	Y	GBM
		M	31	4	NA	B	1.3	D	N	Y	GBM
		M	52	4	NA	S	15	A	N	N	GBM
Kleinschmidt-Demasters et al. [28]	2018	F	27	4	Th	B	10.2	D	NA	NA	GBM
		F	33	3	Th	B	5.1	D	NA	NA	An Astro
		F	72	4	Th	B	3.2	D	NA	NA	GBM
Gao et al. [14]	2018	F	51	4	C	S	6	D	N	N	DMG
Peters et al. [29]	2019	M	39	4	C	S	31	D	Y	Y	GBM
Velz et al. [10]		F	25	4	CT	B	0.4	D	N	N	GBM
Uppar et al. [8]	2019	F	28	4	C	S	0.76	D	N	N	GBM
Goldstein et al. [30]	2019	F	19	4	Th	B	NA	NA	Y	Y	DMG
Schreck et al. [31]	2019	M	31	4	NA	B	4	D	N	N	GBM
		F	38	3	NA	B	25	D	Y	Y	An Astro
Lebrun et al. [25]	2020	NA	NA	4	NA	B	21	A	NA	NA	An Astro
		NA	NA	4	NA	B	27	D	NA	NA	An Astro
		NA	NA	4	NA	B	31	D	NA	NA	An Astro
		NA	NA	4	NA	B	20	D	NA	NA	An Astro
		NA	NA	4	NA	B	43	D	NA	NA	An Astro
Ebrahimi et al. [5]	2020	M	23	4	NA	B	NA	NA	NA	NA	GBM
		F	31	4	NA	B	NA	NA	NA	NA	GBM
		M	58	2	NA	B	NA	NA	NA	NA	Diff Astro
		F	61	3	NA	B	NA	NA	NA	NA	An Astro
		M	73	2	NA	B	NA	NA	NA	NA	Diff Astro
		M	73	4	NA	B	NA	NA	NA	NA	GBM
Dono et al. [32]	2020	F	23	4	NA	S	9.3	D	Y	Y	DMG
		F	33	4	NA	B	34.8	D	Y	Y	DMG
Kamidani et al. [33]	2020	M	32	4	CT	B	1.16	A	Y	Y	DMG
Kraus et al. [34]	2020	M	28	4	C	S	NA	NA	Y	Y	DMG
Aftahy et al. [35]	2023	M	28	4	C	S	NA	NA	Y	Y	DMG
Wang L et al. [36]	2020	M	50	4	C	B	19.87	A	Y	Y	DMG
Handis et al. [37]	2021	F	16	4	CT	B	5	D	Y	Y	DMG
Gu et al. [19]	2021	M	49	4	Th	S	15	A	Y	Y	GBM
		F	39	4	Th	S	13	A	Y	Y	GBM
		F	32	4	C	S	27	A	Y	Y	GBM
		M	65	4	CT	S	NA	A	Y	Y	GBM
		F	27	4	Th	S	26	A	Y	Y	GBM
Yabuno et al. [38]	2021	F	47	4	TL	B	17	D	Y	Y	DMG
		F	46	4	C	B	11	D	Y	Y	DMG
Einsten et al. [39]	2021	F	33	4	Th	B	NA	A	N	Y	DMG
Palpan Flores et al. [40]	2021	M	20	4	TL	S	24	A	Y	Y	DMG
		F	33	4	L	B	NA	NA	NA	NA	GBM
Majzner et al. [41]	2021	NA	25	4	Th	B	20	D	NA	N	DMG
Neth et al. [42]	2022	M	28	4	Th	S	30	D	Y	Y	DMG
		F	48	4	C	S	42	A	Y	Y	DMG
		F	22	4	C	B	6	A	Y	Y	DMG
Horta et al. [43]	2022	F	21	4	Th	B	NA	NA	Y	Y	DMG
Maimati et al. [44]	2022	F	61	4	CT	NA	3	D	N	N	GBM
Our Case	2022	M	25	4	Th	S	20	A	Y	Y	GBM
Bassa et al. [45]	2023	F	31	4	TL	NA	20	A	N	Y	DMG
Chen et al. [46]	2023	M	32	4	TL	S	6	D	Y	Y	DMG
Saluja et al. [47]	2023	M	38	4	TL	S	NA	D	N	Y	DMG
Mo et al. [48]	2023	M	20	4	Th	B	6	D	Y	Y	DMG
		F	23	4	Th	B	18	D	Y	Y	DMG

A: alive; An Astro: anaplastic astrocytoma; B: biopsy; C: cervical tract; CT: cervical-thoracic tract; Th: Thoracic tract; TL: thoracic-lumbar tract; L: Lumbar tract; D: Dead; Diff Astro: diffuse Astrocytoma; DMG: diffuse midline glioma; GBM: glioblastoma; N: number of cases; M: male F: female; NA: not available; FU: follow-up; m: months; S: surgery; RTx: radiotherapy; Ctx: chemotherapy; m: months; d: days; y: yes; n: no. * WHO: according to WHO 2016 for articles before 2022, and according to WHO 2021 for articles after 2022.

## Data Availability

Not applicable.
